# Radiation pneumonitis complicated by *Pneumocystis carinii* in patients with thoracic neoplasia: a clinical analysis of 7 cases

**DOI:** 10.1186/s40880-019-0392-6

**Published:** 2019-08-23

**Authors:** Zhixue Fu, Xu Yang, Nan Bi, Yirui Zhai, Dongfu Chen, Wenqing Wang, Lei Deng, Tao Zhang, Zongmei Zhou, Jun Liang

**Affiliations:** Department of Radiation Oncology, National Cancer Center/National Clinical Research Center for Cancer/Cancer Hospital, Chinese Academy of Medical Sciences and Peking Union Medical College, Beijing, 100021 P. R. China

## Dear editor,

Patients with neoplastic disease may have immunosuppression due to primary disease or related treatment [1]. They have an increased risk of bacterial infections and *Pneumocystis carinii* pneumonia (PCP). Symptomatic radiation pneumonitis (RP) is an important clinical toxic response in patients receiving thoracic radiotherapy. The incidence is 15%–40% in patients receiving concurrent chemoradiotherapy for non-small cell lung cancer (NSCLC) [2]. RP can reduce the quality of life and uncommonly result in oxygen dependence or death. RP complicated by PCP is rarely reported and the clinical characteristics are unclear. Here, we reported 7 cases of thoracic neoplastic patients with RP complicated by PCP treated at the Cancer Hospital, Chinese Academy of Medical Sciences (Beijing, China) between October 2014 to March 2017 to study their clinical characteristics and treatment outcomes (Additional file 1: Table S1).

## Clinical characteristics

In the present study, 4 patients were diagnosed post radiotherapy and 3 during radiotherapy. All patients enrolled were male. Only one of them was a nonsmoker. The median age was 57 years (range, 50–72 years). Of the 7 patients, 3 were diagnosed with lung cancer (stage I to III), 3 with esophageal squamous cell carcinoma (stage II to IV), and 1 with thymus squamous carcinoma (Masaoka stage IV). Additionally, 2 patients had diabetes. One of them also had lower limbs thrombus. One patient had both hypertension and emphysema and 1 had dermatomyositis (DM) that was treated with steroid. PCP was an uncommon, but fatal opportunistic infection in patients with DM. Li et al. [3] reported that PCP should be considered in patients with DM or polymyositis (PM) complicated with interstitial lung disease and receiving cytotoxic agents and corticosteroids.

All patients received thoracic intensity modulated radiation therapy (IMRT) with 6Mv-X ray. Four patients received concurrent chemoradiotherapy, 2 received postchemotherapy irradiation and 1 received irradiation alone. The median values of gross tumor volume (GTV) and plan target volume (PTV) were 32.68 cm^3^ (range, 10.33–140.70 cm^3^) and 361.44 cm^3^ (range, 41.58–725.34 cm^3^). The median volume of both lungs was 3373.87 cm^3^ (range, 2219.8–4333.02 cm^3^). The median proportions of lung V5, V20 and V30 were 54% (range, 42%–70%), 18% (range, 8%–26%) and 12% (range, 5%–20%), respectively. The mean dose of whole lung was 1110.4 cGy (range, 695.5–1495.6 cGy) and that of total radiation was 54 Gy (range, 34–64 Gy). The median fraction dose was 2 Gy (range, 2–4 Gy).

The first symptom of all RP patients was fever. Other symptoms included shortness of breath and increased cough without sputum (two patients with PR developed shortness of breath and one developed increased cough without sputum). All patients were diagnosed with RP by chest computed tomography (CT) scan. Three patients were diagnosed RP during their irradiation with total dose of 34–54 Gy. The other four patients were diagnosed at 7, 16, 25 and 27 days after radiotherapy. All patients received antibiotics and steroids. Three patients underwent 10 mg of dexamethasone injection, two 5 mg of dexamethasone injection and one received 30 mg of oral prednisone, twice per day. All symptoms improved significantly after RP treatment. The RP grade of all patients was grade 2 before the treatment. The second chest CT scan after 10 to 14 days of the RP treatment showed that the RP significant improvement in 5 and stable in 2 patients.

The patients was diagnosed PCP when they received steroids for treated RP. The patient with thymus cancer complicated with DM received 40 mg of oral prednisone daily to treat the DM, and developed fever after 31 days of treatment. The RP was subsequently confirmed by chest CT. PCP was diagnosed after a 10 mg of dexamethasone injection was given for the RP treatment. The median interval time from the beginning of the steroid treatment to the onset of PCP symptoms was 29.5 days (range, 15–39 days) days for all patients. Richards et al. [4] hypothesized that PCP should be considered in patients with atypical RP, whether it was atypical because of clinical course, degree of symptoms, or time-dose factors. In our study, however, RP was diagnosed in patients with typical symptom like fever and clinical course, such as during or within one month of irradiation. As the first symptom of PCP, fever was noted in 6 patients and shortness of breath was observed in 1 patient. Kim et al. [5] reported that the most common symptom of PCP was fever (90.6%), followed by dyspnea (78.1%), cough (56.3%) and sputum (34.4%), for patients with non-Hodgkin lymphoma received chemotherapy. Therefore, careful follow-up for fever must be given during steroid tapered during RP treatment. The median time from the onset of the first symptom of PCP to definite PCP was 7 days (range, 3–9 days) for all patients.

**Fig. 1:**
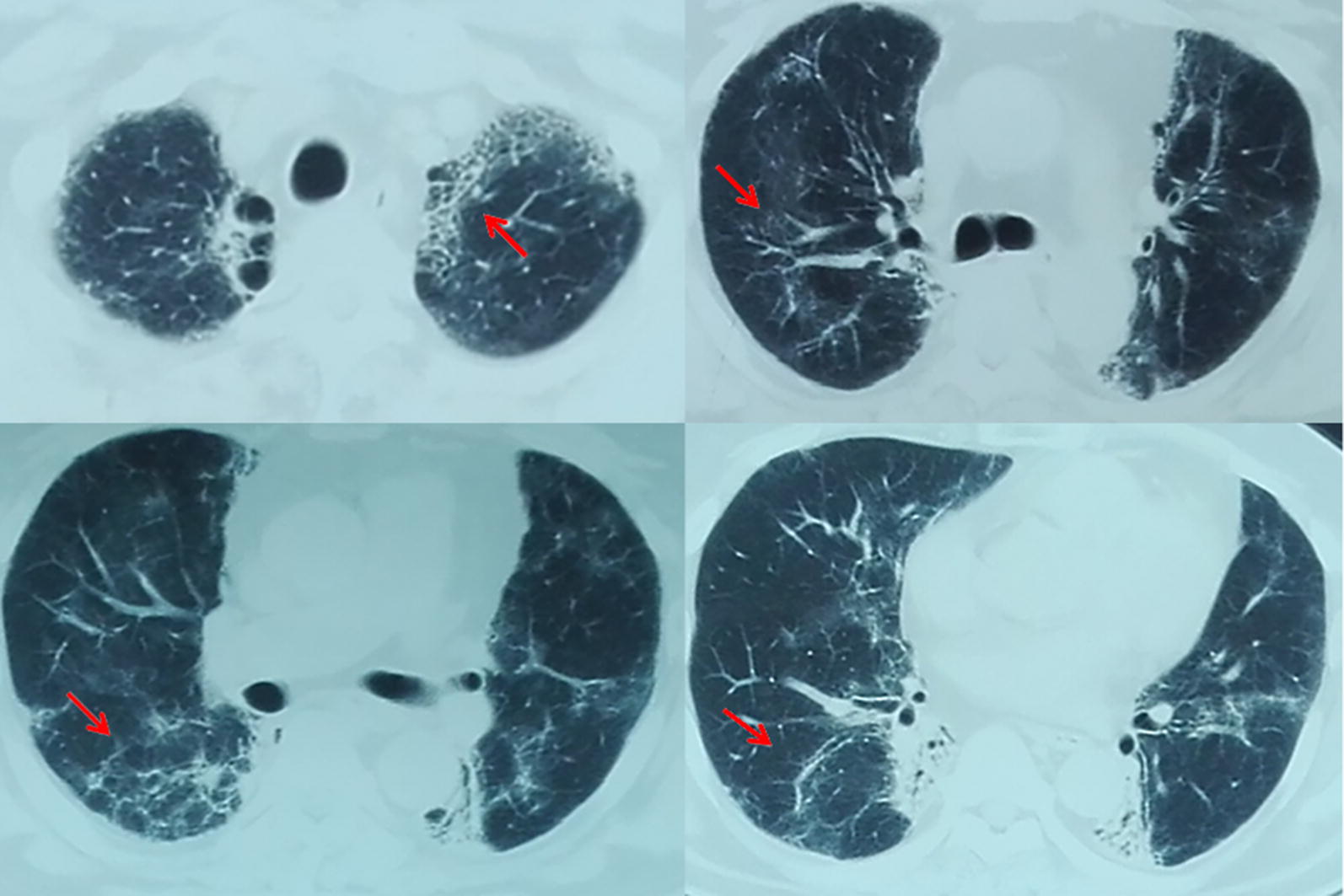
The radiographic findings of one thoracic neoplastic patient with radiation pneumonitis complicated by *Pneumocystis carinii*

All patients showed diffuse pulmonary ground-glass attenuation when RP was diagnosed. There was no relationship between these findings and irradiation fields. The chest CT at 10–14 days after RP treatment revealed a significant improvement in 5 patients and was stable for 2 patients. The chest CT at diagnosis of PCP showed diffuse, bilateral, finely granular or reticular infiltrates in 5 patients. Similar to RP, PCP presents with various atypical radiographic characteristics, including the relationship between the photographic findings and PTV (Fig. 1). This result is controversial compared to other reports. Kim et al. [6] reported 1 case of lung cancer patients who developed PCP while receiving irradiation therapy. They found that the radiation site was spared and appeared as the “photographic negative of post-radiation pneumonia”. Additionally, Forrest et al. [7] reported 2 cases of patients who developed PCP after lung irradiation. They suggested that the irradiated lung tissues could not support the growth of PCP. Panicek et al. [8] hypothesized that PCP infiltrated might avoid the irradiation field. They advised that the finding of “photographic negative of post-radiation pneumonia” was helpful to diagnose PCP in immunocompromised patients. The reasons for the differences between our study and that of other studies may include: (1) our patients received IMRT with more irradiation fields, while patients in other studies underwent two-dimensional radiation therapy only with limited irradiation fields; (2) our patients were diagnosed as RP complicated with PCP, but patients in other studies were diagnosed as PCP pneumonia without RP.

The *Pneumocystis carinii* nucleic acid tests of sputum were positive in 5 patients, and the PCR tests using the specimens of bronchoalveolar lavage (BAL) fluid were positive in 2 patients. One patient had cytomegalovirus (CMV) and Epstein–Barr virus (EBV) infection which were diagnosed by BAL specimen culture. One patient had CMV and candida albicans infection which were diagnosed by sputum culture. One patient had mycoplasma pulmonis infection. The patient with DM also had dermal candida albicans infection. Immunofluorescence assay was performed to detect *Pneumocystis jirovecii* using human and rodent pneucysts- and trophozoites-associated monoclonal antibodies. PCP was difficult to diagnose for several reasons in the past: (1) examination of sputum samples were usually inconclusive; (2) alveolar lavage might detect PCP in few patients; (3) although lung biopsy samples are best for detecting PCP, the sampling method increases the incidence of related complications.

## Treatment

**Fig. 2:**
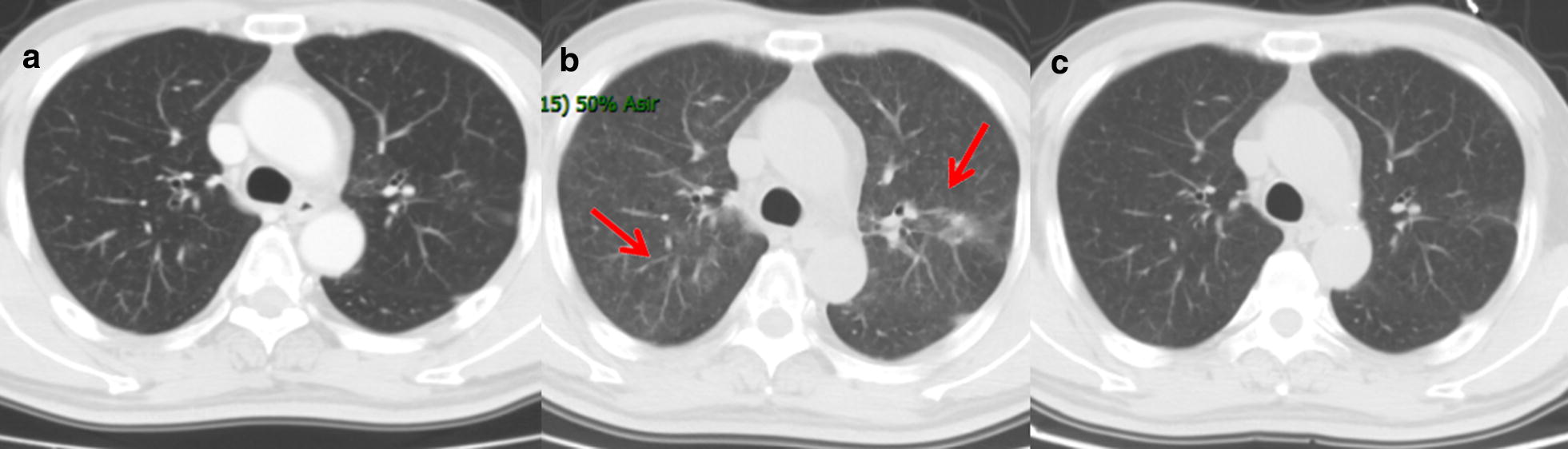
The radiographic findings of one patient with radiation pneumonitis complicated by *Pneumocystis carinii* for three courses. **a** The CT image acquired before irradiation therapy. **b** The CT image acquired when RP complicated by PCP was diagnosed. **c** The CT image acquired after TMP-SMX treatment. *RP* radiation pneumonitis, *PCP Pneumocystis carinii* pneumonia, *TMP–SMX* trimethoprim–sulfamethoxazole

All of the patients we studied received trimethoprim-sulfamethoxazole (TMP-SMX) at therapeutic dose and steroids continuously (Fig. 2). Six patients with PCP were treated with TMP-SMX for 2–3 weeks, after that their sputum culture results turned PCP-negative. Only one patient died of severe pulmonary anaerobic infection 1 month after the diagnosis of PCP. TMP-SMX has achieved great success in inhibiting PCP by inhibiting folate synthesis. However, it is unclear whether anti-PCP prophylaxis is necessary for patients with immunosuppression. Sepkowitz et al. [1] hypothesized that prophylaxis with TMP-SMX was highly effective in preventing PCP and was associated with reduced mortality in patients with neoplastic disease. Colby et al. [9] described that the development of definite PCP was not associated with a poor prognosis. Thus, as long as the patients were cautiously observed, it was not too late to start anti-PCP therapy after the symptoms of PCP manifest. All of the PCP patients we studied were successfully treated with TMP-SMX. However, we thought that prophylaxis with TMP-SMX might not be necessary for patients with PCP. Walzer et al. [10] thought that successful treatment depended upon early recognition of PCP.

## Survival outcomes

The last follow-up time was on May 1, 2017, with the median follow-up time of 15 months (range, 5–21 months). Three patients of our study died, including one esophageal cancer patient who survived for 3.5 months died of severe pulmonary infection 1 month after the diagnosis of PCP, one lung cancer patient with 21 months survival time died of local progression, and one with 16 months survival time died of distance metastases. None of our patients died of PCP. One patient died of severe pulmonary infection with anaerobion. The others have survived for 5–20 months.

In summary, during the use of steroids for treating RP, patients with thoracic neoplasia often develop fever before the diagnosis of PCP. The median interval time from the beginning of the steroid treatment to the onset of PCP symptoms was 1 month. Steroids and TMP-SMX were effective treatment for treating PCP.

## Additional file

**Additional file 1: Table S1.** Clinical characteristics of 7 thoracic neoplastic patients with radiation pneumonitis complicated by *Pneumocystis carinii*.

## Data Availability

The datasets used and/or analyzed during the current study are available from the corresponding author on reasonable request.

## Abbreviations

PCP: *Pneumocystis carinii* pneumonia
RP: radiation pneumonitis
NSCLC: non-small cell lung cancer
DM: dermatomyositis
PM: polymyositis
IMRT: intensity modulated radiation therapy
GTV: gross tumor volume
PTV: plan target volume
CT: computed tomography
BAL: bronchoalveolar lavage
CMV: cytomegalovirus
EBV: Epstein–Barr virus
TMP–SMX: trimethoprim–sulfamethoxazole
