# Supplementary material for: Radiation pneumonitis complicated by Pneumocystis carinii in patients with thoracic neoplasia: a clinical analysis of 7 cases
Source: Cancer Commun (Lond). 2019 Aug 23;39:47. doi: 10.1186/s40880-019-0392-6 (PMC6708139; doi:10.1186/s40880-019-0392-6)
Supplement: Supplementary file 1 — Additional file 1: Table S1. Clinical characteristics of 7 thoracic neoplastic patients with radiation pneumonitis complicated by Pneumocystis carinii. [file 40880_2019_392_MOESM1_ESM.docx]

Table S1. Clinical characteristics of 7 thoracic neoplastic patients with radiation pneumonitis complicated by pneumocystis carinii.

| **Characteristics** | **Patient #1** | **Patient #2** | **Patient #3** | **Patient #4** | **Patient #5** | **Patient #6** | **Patient #7** |
| --- | --- | --- | --- | --- | --- | --- | --- |
| **Gender** | Male | Male | Male | Male | Male | Male | Male |
| **Age (years)** | 58 | 72 | 56 | 50 | 67 | 53 | 56 |
| **Smoking history** | Yes | Yes | Yes | No | Yes | Yes | Yes |
| **Complication** | None | Hypertension and emphysema | Diabetes | Dermatomyositis | Diabetes and lower limbs thrombus | None | None |
| **Diagnose** | Right lung cancer | Esophageal cancer | Left lung cancer | Thymus cancer | Left lung cancer | Esophageal cancer | Esophageal cancer |
| **Pathology** | SCC | SCC | Neuroendocrine carcinoma | SCC | SCC | SCC | SCC |
| **Cancer stage** | T1N0M0 | T4N1M0 | T2N3M0 | Masaoka stage Ⅳ | T3N1M0 | T2N1M0 | T3N1M1 |
| **RT type** | IMRT | IMRT | IMRT | IMRT | IMRT | IMRT | IMRT |
| **RT Dose** | 60Gy/4Gy/15f | 34Gy/2Gy/17f | 54Gy/2Gy/27f | 54Gy/2Gy/27f | 64Gy/2Gy/32f | 47Gy/2.14Gy/22f | 59.92Gy/2.14Gy/28f |
| **Type of therapy** | RT alone | CRT | Postchemotherapy RT | Postchemotherapy RT | CRT | CRT | CRT |
| **CT regimen** | NA | PTX+DDP | Vp-16+DDP | PTX+DDP | Vp-16+DDP | PTX+DDP | PTX+DDP |
| **First symptom of RP** | Fever | Fever | Fever | Fever | Fever | Fever | Fever |
| **Treatment of RP** | LVFX+Dex | Tien+Dex | Tien+Dex | Tien+Pred | LVFX+Dex | LVFX+Dex | LVFX+Pred |
| **Response of RP** | CR | SD | SD | PR | PR | PR | PR |
| **First Symptom of PCP** | Shortness of breath | Fever | Fever | Fever | Fever | Fever | Fever |
| **Interval time from RP to PCP (days)** | 39 | 32 | 19 | 34 | 15 | 27 | 16 |
| **Time of PCP diagnosis* (days)** | 7 | 8 | 7 | 7 | 9 | 7 | 3 |
| **Diagnosis method of PCP** | Sputum | Sputum | Sputum | BAL | BAL | Sputum | Sputum |
| **Infection-related complications of PCP** | None | CMV and Candida albicans | Mycoplasm pulmonis | Dermal candida albicans | CMV, EBV | None | None |
| **Treatment of PCP** | TMP-SMX | TMP-SMX | TMP-SMX | TMP-SMX | TMP-SMX | TMP-SMX | TMP-SMX |

* The time from the first symptom of PCP developed to PCP diagnosis

Abbreviations: SCC, squamous cell carcinoma; T, tumor; N, nodal metastasis; M, metastasis; RT, radiotherapy; IMRT, intensity modulated radiation therapy; CRT, concurrent chemoradiotherapy; CT, chemotherapy; PTX, paclitaxel; DDP, cisplatin; RP, radiation pneumonitis; LVFX, levofloxacin; Dex, dexamethasone; Tien, tienam; Pred, prednisone; CR, complete response; SD, stable disease; PR, partial response; PCP, pneumocystis carinii pneumonitis; BAL, bronchoalveolar lavage; CMV, cytomegalovirus; EBV, epstein-barr virus; TMP-SMX, trimethoprim-sulfamethoxazole; NA, not applicable.
